# Referral thresholds for an integrated learning disability eye care pathway: a consensus approach

**DOI:** 10.1038/s41433-021-01516-y

**Published:** 2021-04-08

**Authors:** Rachel F. Pilling, Lisa Donaldson, Marek Karas, R. Jane Leitch, Howard Bunting, Ravi Naru, Gordon Ilett

**Affiliations:** 1 School of Optometry and Vision Science, University of Bradford, BD7 1DP Bradford, UK; 2 Department of Ophthalmology, Bradford Royal Infirmary, Bradford Teaching Hospitals NHS Trust, Bradford, BD9 6RJ UK; 3 SeeAbility, Newplan House, 41 East Street, Epsom, Surrey KT17 1BL UK; 4 School of Optometry & Vision Sciences, Cardiff University, CF24 4HQ Cardiff, UK; 5 Department of Ophthalmology, ESTH University Hospitals Trust St Helier Hospital Wrythe Lane, Carshalton, SM5 1AA UK; 6 Department of Ophthalmology, Princess Royal University Hospital, Orpington, Kent BR6 8ND UK; 7 Ravi Naru Opticians, Bradford, BD18 3TJ UK; 8 Linklater & Warren Opticians, Sevenoaks, TN13 1LP UK

**Keywords:** Epidemiology, Vision disorders

## Abstract

**Introduction:**

Local Optometric Support Unit (LOCSU) have published their refreshed clinical pathway for eye care for people with a learning disability. The document sets out the adjustments to practice that a community optometrist might make in order to provide optimal care for a person with learning disability attending a primary eye care assessment. The pathway specifically points to the need to retain patients in primary care where appropriate and ‘reduce the number of people with learning disability who are inappropriately referred into the Hospital Eye Service (HES).’ Pivotal to this refreshed pathway is the integration with secondary care, with local arrangements to facilitate referral and hospital management where appropriate. There are few ophthalmologists nationally who frequently encounter patients with a learning disability in their hospital practice and knowing where to start when creating referral criteria or KPIs may create a barrier to services becoming established. In order to address this gap in experience, we set about developing a set of consensus statements regarding referral thresholds for ocular conditions commonly encountered in adults with learning disability.

**Method:**

A series of video interviews were undertaken with eye health professionals with a range of experience in eye care for people with learning disability. Each contributor commented on the usability and clarity of each element of the referral criteria. In addition, each contributor was asked to express the overriding principles by which they make decisions regarding referral thresholds for patients with learning disability. These were collated into the final document which was circulated and agreed by all participants.

**Results:**

A table setting out referral thresholds for commonly encountered eye conditions in adults with learning disabilities is presented.

**Conclusion:**

We have presented a succinct set of consensus statements relating referral thresholds for common presentations of visual problems in adults with learning disability in the UK distilled from the collective experience of a group of eye health professionals. The intention was not to present a comprehensive review of management of each condition. Rather, the consensus statements may form the starting point from which each area could develop locally agreed criteria, as is suggested by the LOCSU pathway guidance.

## Introduction

People with learning disability in England number ~1.2 million [1]. They face significant health inequalities [2]: people with a learning disability are ten times more likely to have vision problems than the rest of the population [3]; over fifty percent of those with learning disability who died prematurely had a visual problem [4]. Compounding this increase in prevalence of eye disease is research showing that people with learning disability are less likely to have access to eye care than the rest of the population [5, 6]. There are several reviews in the ophthalmic and optometric literature which detail the prevalence and incidence of specific conditions in both paediatric and adult populations with learning (or intellectual) disability [5, 7–11]. People with learning disability have a right to equal standards of health care by law [12]. Several groups, including the Royal College of Ophthalmologists, have called for changes to policy and the whole eye care pathway to allow patients to access services and receive equality of care [13, 14].

In order to address this, the Local Optometric Support Unit have published their refreshed clinical pathway for eye care for people with a learning disability [15]. The document sets out the adjustments to practice that a community optometrist might make in order to provide optimal care for a patient with learning disability attending routine primary eye care. A key difference with this refreshed guideline compared with its predecessor is the ambitious aim to improve integration between primary eye care and hospital eye services.

By providing services through the LOCSU scheme, the optometrist is agreeing to use their professional judgement and reasoning, drawing on their training and relationship with hospital eye services to determine if the patient would benefit from further assessment by the hospital eye services.

When a local pathway is newly established, it is anticipated that for the majority of patients, the optometrist will be meeting the patient for the first time and there will be little in the way of previous records. Few patients with learning disability achieve ‘normal’ vision [9, 16–19] and it is likely that a high number of ocular abnormalities will be detected [9, 20–22]. Some patients will require hospital eye services for assessment, treatment or registration for sight impairment; but for others with longstanding or congenital ocular abnormalities, referral into the hospital eye service will add little value and be stressful for the patient and carer.

Within the LOCSU pathway lie key performance indicators (KPIs) which are to be reported quarterly. These include the percentage of patients referred from the LD community eye care service into secondary eye care, the target for which is set locally. In order to facilitate this, dialogue between primary and secondary care will need to take place to establish what referrals would be considered to add value to the patient or carer.

There are few ophthalmologists nationally who frequently encounter patients with a learning disability in their hospital practice and knowing where to start when creating referral criteria or KPIs may create a barrier to services becoming established. In order to address this gap in experience, we set about developing a set of consensus statements regarding referral thresholds for ocular conditions commonly encountered in adults with learning disability.

## Method

Eye Health Professionals known to be involved in the community and hospital management of adults with learning disability were approached and invited to participate. The group comprised two specialist learning disability optometrists, two community optometrists and two consultant ophthalmologists. A series of telephone and video interviews were undertaken by one of the authors (RP). A baseline for referral thresholds was drawn up, based on those previously agreed by consensus for the NHS England Special School Eye Care Programme (unpublished). Each member of the group commented on the usability and clarity of each element of the referral criteria and any additional research or evidence which might support the referral threshold. In addition, each contributor was asked to express the overriding principles by which they make decisions regarding referral thresholds for patients with learning disability. Individual comments were collated into the final document which was circulated and agreed upon by all participants.

## Results

### Overriding principles

The referring optometrist should consider each of these questions when determining if the patient would benefit from referral into the Hospital Eye Service (HES).

- Are there any new findings – has the patient previously been seen by HES?
- Has there been a change in function noted by the patient, carers, family?
- Is this likely to impact the patient’s social function and activities of daily living?
- It is a stable or progressive condition?
- Is there any treatment that could be started in the community which would not necessitate referral into the HES? (including advice, spectacles, rehabilitation, low vision appliances.)

The table below lays out the suggested referral thresholds and accompanying notes for common ocular conditions found in patients with learning disability.

**Table 1:** Referral Thresholds for common clinical findings in patients with learning disability.

Element	Referral threshold	Notes
Visual Function		
Visual Acuity/Functional Visual Assessment	Recent change in visual function Distance vision worse than 6/19* (WHO low vision definition [23]) Near vision worse than 6/19* N12 or equivalent *after adaptation to refractive correction	If the patient has previously been documented as having poor vision and there is no change in function, then referral may not be required. Visual Function may be measured using Bradford Visual Function Box [24]. No interest in an object smaller than 30 mm target at 50 cm would trigger referral Consider referral to Eye Clinic Liaison Officer, Low Vision Services and Sensory Services team Consider referral to HES for sight impairment registration where appropriate
Contrast Sensitivity (CS)		There is no specific referral threshold: however, the impact of impaired CS on a patient’s ability to access visual materials is a key factor that should be included in any report or feedback and taken into consideration when considering eligibility for sight impairment certification.
Colour vision	No referral required for patients with abnormal colour vision unless known to have been acquired or associated with other change in visual function/optic disc changes.	
Refraction		
Refractive Error	Refractive error should be managed in community	Appropriate advice should be offered to patients/carers where high refractive error may be associated with complications eg signs of retinal detachment, angle closure glaucoma. High refractive error alone does not warrant HES referral.
	Astigmatism if progressive >1D per year	Referral for keratoconus investigation may be appropriate where there is progressive astigmatism/eye rubbing
Presbyopia & Accommodation	Development of presbyopia should be considered in patients of appropriate age.	This should be managed in the community with NV specs/bifocal/varifocal [25] It should be noted that lack of accommodation is common in patients with learning disability [26–28].
Red reflex	Reduced red reflex—refer if new finding	Consider if cataract is impacting on visual function prior to referral. Consider use of Visual Symptoms in Learning Disability (VSLD) [29] to document visual function prior to referral to allow monitoring of impact on vision
Incomplete Examination	Consider referral if a specific concern is raised from the patient or carer, from a change in visual function or new ocular symptoms. Consider referral if the patient may benefit from an orthoptist assessment of vision.	LOCSU pathway [15] states that several attempts will be made by the community optometrist over a prolonged period to complete the assessment. In the absence of new symptoms or specific pathology, referral need not be made purely to complete the examination It is rare that examination under anaesthetic would be offered in the absence of specific concerns or new signs/symptoms
Lids		
Ptosis/Entropion/Ectropion	Refer if new finding or impairing patient’s visual function.	Consider offering advice re interim measures for en/ectropion eg gel/ointment. Consider video/telephone & photo consultation with HES.
Chalazion	Refer if atypical features and/or not responding to community management measures	
Trichiasis	Refer if symptomatic and evidence of corneal involvement	Consider epilation in the community
Anterior segment		
Red eyes/blepharitis	Refer if not responding to community based measures. Refer if patient photophobic and/or evidence of corneal involvement	Consider utilising Minor Eye Condition Scheme (MECS) [30] if symptoms are acute. Consider video or telephone consultation with patient/carers to discuss second level management options. Dry eye/chronic eye rubbing may require medication to manage and/or investigation for keratoconus (especially in Down’s syndrome patients [31])
Microphthalmos, coloboma	Refer if associated with reduced vision eligible for sight impairment registration	Consider video or telephone consultation with patient/carers to explain condition and ensure associated medical conditions have been investigated in childhood. Consider genetic testing or counselling if patient or carer would like to explore this. Community monitoring for glaucoma may also be appropriate after discussion with HES
Corneal opacity/irregularity	Refer if previously undiagnosed or progressive. Refer if Scissor reflex present on retinoscopy.	Scissor reflex is sensitive sign for early keratoconus [31], especially in the Down’s syndrome population, and should prompt referral.
Glaucoma		
Raised intraocular pressure (IOP)	Refer as per NICE guidelines [32].	If a local referral refinement scheme is in operation, this should be utilised.
Unable to obtain IOP measurement	Referral is not necessary unless there are associated risk factors (patient over age 40 and first degree relative with POAG, other anterior segment abnormalities associated with raised IOP).	Attempts should be made to engage patients over several visits using different IOP tools (eg Icare) to obtain IOP.
Narrow angles Van Herrick	Discussion with HES to determine if additional risk factors associated with Angle Closure Glaucoma (ACG).	Unless patient high hypermetropia or other anterior segment abnormality eg microphthalmia, typically reasonable to offer patient and carers advice regarding signs and symptoms of ACG.
Atypical Visual Fields	Visual field deficits observed on confrontation field testing should be discussed with the HES to consider further investigation	Formal Visual Fields (eg Humphrey) should only be performed if there is a specific concern. Results may be difficult to interpret if the patient has problems engaging with the test procedure. Consider also referral to ECLO, Low vision or for sight impairment registration.
Fundus		
Inability to perform fundal examination due to poor engagement	If there is no change to visual function, then no referral necessary but attempts to assess fundus should be made each year.	Specific warnings about retinal detachment symptoms should be offered to patients/ carers where there is a previous history of retinopathy of prematurity or retinal detachment
Small optic disc	Refer only if not previously investigated in childhood	
Pale optic disc	Refer only if not previously documented or change in visual function	
Swollen optic disc	Refer, bearing in mind prevalence of Crowded disc appearance associated with hypermetropia	
Diabetic Retinopathy	Referral of patients with R3, R2 or M1 disease [33]	Check patient is under Diabetic Eye Screening Programme. Consider reasonable adjustments to support patient and carer to access the DESP [34, 35].
Drusen/AMD	If evidence of wet AMD, utilise local referral pathways as per NICE guidance [36].	For dry AMD consider ECLO/Low vision referral/support organisations. Liaise with HES to determine most appropriate time/location for patient assessment, highlighting patients LD and what reasonable adjustments may be useful.
Ocular Motility		
Nystagmus	Refer if symptomatic, atypical features, vertical nystagmus or new finding.	Horizontal or manifest latent nystagmus is common in adults with learning disability. Consider discussion with HES if patient may be eligible for sight impairment registration. Refer to ECLO and low vision for support where appropriate. Note that newly acquired nystagmus will be associated with oscillopsia and the patient will be symptomatic with a change in behaviour.
Strabismus	New or changing ocular deviation/squint. Patients who express concern about their squint should be referred for consideration for ocular alignment (squint) surgery.	Adults with learning disability may assume a “relaxed” position of exotropia when not visually engaged and then realign for short periods when searching for an object. Long-standing esotropia is a common finding and may be associated with reduction in abduction [37]. Using photographs can be useful for establishing a change or new presentation. Appearance of new exotropia can indicate a loss of vision; appearance of a new esotropia may indicate blocked shunt or decompensated hydrocephalus if the deviation is larger in the distance than near.
Stereoacuity	No referral required for adults with abnormal stereoacuity	

### Conclusion

The aim of the LOCSU pathway is to raise awareness of, and improve access to, eye care for people with learning disability. It offers an opportunity to identify those patients for whom intervention or support can be offered, in the community or hospital eye service; be that spectacles, low vision support, baseline assessment from which future change can be measured or progressive, treatable eye disease.

In order for a pathway to improve access to eyecare to be successfully implemented, it is necessary to raise awareness of both the likely barriers that patients, carers and eye health professionals may encounter, and highlight existing guidance, research and good practice in overcoming these barriers. Most ophthalmologists in the UK have occasional exposure to patients with learning disability and will be seeking a place from where to begin conversations with their local optometric committees.

We have presented a succinct set of consensus statements relating referral thresholds for common presentations of visual problems in adults with learning disability in the UK distilled from the collective experience of a group of eye health professionals (Table 1). The intention was not to present a comprehensive review of management of each condition. The consensus statements may form the starting point from which each area could develop locally agreed criteria, as is suggested by the LOCSU guidance [15]. A future extension of these consensus statements may be to develop an accepted minimum dataset for patients seen within the LOSCU learning disability pathway. This would enable the development of population-based data, including prevalence of ocular conditions in the learning disability population, enable robust evidence collection on the impact of interventions and point to areas for future research. The recent expansion into telephone or video consultations which have proved successful in many areas offers alternative delivery options which may be more accessible to patients and carers and lend opportunity as a reasonable adjustment to face-to-face hospital visits.

## Summary

### What was known before

- Adults with learning disability have a higher prevalence of sight impairment than the general population, yet are less likely to access primary eye care.
- The key to a successful community eye care service for adults with learning disability is close integration with secondary eye care.

### What this study adds

- Referral thresholds indicating which patients would benefit from referral into secondary care are presented.
- Local Eye Health Networks or Integrated Care Systems may consider using these as a starting point when commissioing a service.

## References

[1] Mencap. Research and Statistics ‘How common is a learning disability’, 2020.

[2] Emerson E, Baines S. Health Inequalities and people with learning disabilities in the UK: improving health and lives: learning disabilities observatory, 2010.

[3] Emerson E, Roberston J. The estimated prevalence of visual impairment among people with learning disabilities in the UK: improving health & lives: learning disabilities observatory, 2011.

[4] Heslop P, Blair P, Fleming P, et al. Confidential Inquiry into premature deaths of people with learning disabilities (CIPOLD). In: Health Do, ed.: Norah Fry Research Centre, 2013.

[5] Donaldson LA, Karas M, O’Brien D (2019). Findings from an opt-in eye examination service in English special schools. Is vision screening effective for this population?. PLoS One.

[6] Li J, Wong K, Park AS (2015). The challenges of providing eye care for adults with intellectual disabilities. Clin Exp Optom.

[7] Salt A, Sargent J (2014). Common visual problems in children with disability. Arch Dis Child.

[8] Evenhuis HM (1995). Medical aspects of ageing in a population with intellectual disability: I. Visual impairment. J Intellect Disabil Res.

[9] Woodhouse JM, Adler PM, Duignan A (2003). Ocular and visual defects amongst people with intellectual disabilities participating in Special Olympics. Ophthalmic Physiol Opt.

[10] van Splunder J, Stilma JS, Bernsen RM (2004). Prevalence of ocular diagnoses found on screening 1539 adults with intellectual disabilities. Ophthalmology.

[11] Warburg M (2001). Visual impairment in adult people with intellectual disability: literature review. J Intellect Disabil Res.

[12] UK Public General Acts. Equality Act, 2010.

[13] Ophthalmologists RCo. Ophthalmic service guidance: eye care for adults with learning disabilites 2015. https://www.rcophth.ac.uk/wp-content/uploads/2015/09/Eye-Care-Services-for-Adults-with-Learning-Disabilities.pdf.

[14] Starling S, Willis A, Dracup M (2006). Right to sight: accessing eye care for adults who are learning disabled. J Intellect Disabil.

[15] LOCSU. Eye care pathway for people with learning disabilities: service delivery proposal, 2020.

[16] Zahidi AA, Vinuela-Navarro V, Woodhouse JM (2018). Different visual development: norms for visual acuity in children with Down’s syndrome. Clin Exp Optom.

[17] Chokron S, Kovarski K, Zalla T (2020). The inter-relationships between cerebral visual impairment, autism and intellectual disability. Neurosci Biobehav Rev.

[18] van Splunder J, Stilma JS, Bernsen RM (2006). Prevalence of visual impairment in adults with intellectual disabilities in the Netherlands: cross-sectional study. Eye.

[19] Van Splunder J, Stilma JS, Evenhuis HM (2003). Visual performance in specific syndromes associated with intellectual disability. Eur J Ophthalmol.

[20] Pilling RF, Outhwaite L (2017). Are all children with visual impairment known to the eye clinic?. Br J Ophthalmol.

[21] Woodhouse JM, Davies N, McAvinchey A (2014). Ocular and visual status among children in special schools in Wales: the burden of unrecognised visual impairment. Arch Dis Child.

[22] Uzdrowska M, Woodhouse JM (2016). Visual defects in special olympics participants from Europe. Clin J Sport Med.

[23] World Health Organisation. World Health Organisation Priority Eye Diseases [https://www.who.int/blindness/causes/priority/en/index4.html. Accessed 11 October 2020.

[24] Pilling RF, Outhwaite L, Bruce A (2016). Assessing visual function in children with complex disabilities: the Bradford visual function box. Br J Ophthalmol.

[25] Al-Bagdady M, Stewart RE, Watts P (2009). Bifocals and Down’s syndrome: correction or treatment?. Ophthalmic Physiol Opt.

[26] Anketell PM, Saunders KJ, Gallagher SM (2018). Accommodative function in individuals with autism spectrum disorder. Optom Vis Sci.

[27] Doyle L, Saunders KJ, Little JA (2016). Trying to see, failing to focus: near visual impairment in Down syndrome. Sci Rep.

[28] Andersson S, Persson EK, Aring E (2006). Vision in children with hydrocephalus. Dev Med Child Neurol.

[29] Rostron E, Rawse C, Piling RF (2018). Validation of VSLD questionnaire in patients with learning disabilities undergoing cataract surgery. Eye.

[30] LOCSU. Minor Eye Conditions Service (MECS) Pathway, 2016.

[31] Little JA, Woodhouse JM, Saunders KJ (2009). Corneal power and astigmatism in Down syndrome. Optom Vis Sci.

[32] National Institute for Health and Care Excellence. Glaucoma: diagnosis and management NICE guideline [NG81 2017 [https://www.nice.org.uk/guidance/ng81. 31909934

[33] Public Health England. Diabetic eye screening standards: Public Health England, 2020.

[34] Pilling RF (2014). Diabetic eye screening in people with learning disabilities: Improving access. J Diabetes Nurs.

[35] Pilling R. Screening for diabetic retinopathy in adults with learning disability: current uptake and adjustments to facilitate equality of access. Br J Learn Disabil. 2014;43 [published Online First: 10.1111/bld.12088]

[36] National Institute for Health and Care Excellence. Age-related macular degeneration NICE guideline [NG82], 2018. 29400919

[37] Woodhouse JM, Griffiths C, Gedling A (2000). The prevalence of ocular defects and the provision of eye care in adults with learning disabilities living in the community. Ophthalmic Physiol Opt.

